# The Role of Trusted Adults in Shaping Adolescent Health Information Access and Mental Wellbeing in Urban India

**DOI:** 10.21203/rs.3.rs-7093966/v1

**Published:** 2025-08-19

**Authors:** Anupam Sharma, Priya Shankar, Ricky Sharma, Nelabh Krishna, Nanditha Jayakumar, Samruddhi Nalawade, Avani Doshi, Aruna Nannapaneni, Vandana Krishnan, Mohammed Danish, Kym Ahrens, Sarah Golub, Rajib Dasgupta, Latha Ravichandran, Padma Srikanth, Sarala Premkumar, Jason M. Nagata, Vaishali Keswani, Isabel Allen, Julia Seaman, Harish Pemde

**Affiliations:** Adolescent Health Champions (AHC); Johns Hopkins University; Adolescent Health Champions (AHC); Adolescent Health Champions (AHC); Adolescent Health Champions (AHC); Adolescent Health Champions (AHC); Adolescent Health Champions (AHC); Adolescent Health Champions (AHC); Adolescent Health Champions (AHC); Adolescent Health Champions (AHC); University of Washington; Seattle Children’s Hospital; Jawaharlal Nehru University; Sri Ramachandra Institute of Higher Education and Research; Sri Ramachandra Institute of Higher Education and Research; Sri Ramachandra Institute of Higher Education and Research; University of California, San Francisco; Kalawati Saran Children’s Hospital; Bay View Analytics; Bay View Analytics; Lady Hardinge Medical College

## Abstract

**Background:**

Adolescents in urban India face significant challenges in accessing healthcare and reliable health information, particularly during crises such as the COVID-19 pandemic. However, the role of trusted adults in shaping adolescent health remains underexplored.

**Objectives:**

This study examined healthcare access, health information exposure, and the influence of trusted adults among urban Indian adolescents during the COVID-19 pandemic.

**Methods:**

A cross-sectional online survey was conducted with 790 adolescents aged 10–19 years across urban India during the second wave of COVID-19 (May–October 2021). The survey assessed mental health, healthcare access, sources of health information, and the presence of trusted adults. Multivariable logistic regression models were used to examine associations between trusted adults and key health (and related) outcomes, adjusting for age, gender, and type of school.

**Results:**

Most adolescents (85.3%) reported having a trusted adult. Adolescents with a trusted adult had significantly higher odds of accessing a healthcare provider (AOR = 1.98, *p* = 0.001) and receiving information on mental health (AOR = 2.38, *p* < 0.001), nutrition (AOR = 1.79, *p* = 0.005), and COVID-19 (AOR = 1.91, *p* = 0.003). The presence of a trusted adult was strongly associated with lower psychological distress during (AOR = 0.40, *p* < 0.001) and before (AOR = 0.43, *p* < 0.001) the pandemic. No significant association was found with telehealth use or access to sensitive health information such as sexual and reproductive health.

**Conclusions:**

Trusted adults play a critical protective role in adolescent health by improving healthcare access and supporting mental wellbeing. Integrating trusted adult frameworks into adolescent health programs may help strengthen supportive environments across homes, schools, and communities.

## 1 Introduction

India is home to over 253 million adolescents, the largest adolescent population in the world [1]. This cohort, comprising individuals between the ages of 10 and 19, represents a significant demographic in the country’s public health landscape. Urban India, with its rapid expansion and demographic complexity, is increasingly the site of heightened inequalities in access to healthcare, particularly among adolescents. Despite the presence of public health infrastructure, adolescents in urban settings, especially those from economically or socially marginalized groups, often face considerable barriers in accessing health services that are confidential, youth-sensitive, and of high quality [2, 3].

The Indian adolescent health policy framework indeed acknowledges these issues. Launched in 2014, the Rashtriya Kishore Swasthya Karyakram (RKSK) had the broad objective of expanding adolescent health beyond a reproductive focus and into six other vital areas, viz, nutrition, mental health, substance abuse, and violence etc. [4]. It conceived of adolescent-friendly clinics and peer counsellors as key mechanisms for the achievement of this objective. However, on the ground, implementation challenges remain with low adolescent attendance, infrequent outreach, and health workers not trained adequately for adolescent engagement [5]. Its design continues to focus on infrastructure and service delivery rather than on relational factors like trust, comfort, and emotional safety, which can either deter or encourage an adolescent to ask for help for oneself.

While much of the discourse around adolescent health has traditionally focused on service availability and infrastructural gaps [3], recent global literature suggests that such an approach is incomplete. Emerging empirical work highlights the important role of social relationships and trust in shaping how adolescents access and interpret health-related information and services. In particular, the presence of a trusted adult, defined broadly as any supportive, non-judgmental adult figure in an adolescent’s life, has been associated with improved health-seeking behaviors, better mental health outcomes, and more reliable access to credible health information [6, 7]. Trusted adults can foster an environment of psychological safety that enables adolescents to express vulnerability and seek help without fear of judgment or rejection [8, 9].

Theories of emerging adulthood stress the complex nature of adolescents’ development and behavior, and suggests that as young people transition into adulthood, they face significant decisions about their education, health, careers, relationships, and values [10]. While these years involve making essential and crucial choices shaping their social and economic futures, developmental research also shows that this is a period when many begin distancing themselves from traditional sources of guidance. Although family, particularly parents, continues to play an important role, young people often seek greater independence during this phase [11]. While for some adolescents, parents could still serve as the point of contact for any health-related advice, adolescents may also seek identity and belonging beyond their immediate families, that is, they often form meaningful relationships with adults in broader social environments [12]. For some, especially those lacking social or cultural capital, consistent access to such relationships may be limited [13]. Nevertheless, the presence of trusted adults is a common and important part of adolescent development.

Trusted adults are often defined by the role they play in offering emotional security and guidance [14]. They are individuals whom adolescents are willing to trust and be vulnerable with, those perceived as reliable, competent, honest, and invested in their well-being, independent of any formal authority or disciplinary power [15]. Importantly, trusted adults could also be typically nonparental figures, such as teachers, extended relatives, coaches, youth workers, religious figures, family friends, or neighbors, who help fill the gap when adolescents seek support outside their family unit [16]. McNeely and Falci (2004) [17] found that school-based adults, teachers in particular, could also serve as “social buffers” that lower maladaptive health outcomes.

In the Indian context, there are only a few studies which address the role of such protective figures in adolescent lives. For instance, one study from India found that open communication between parents and daughters, particularly around school performance, friendships, and personal matters, was linked to a lower likelihood of early marriage among girls [18]. Moreover, such discussions were associated with more collaborative decision-making in selecting a marriage partner, rather than parents making the choice alone [18]. The Youth in India: Situation and Needs 2006–07 study [19] also showed that parent-child communication in India, especially on sensitive issues like puberty, sex, and reproductive health, remains limited, largely shaped by cultural taboos, gender norms, and regional differences. Communication on general topics such as education and behavior was reportedly more common. Discussions around puberty were highly gendered and focused mostly on girls, with mothers assuming responsibility but often deferring to other female relatives due to embarrassment. Fathers rarely discussed these topics with daughters and communicated only minimally with sons. Conversations about sex, pregnancy, and STIs were even more restricted, with many parents believing such topics should not be addressed until after marriage. However, despite prevalent discomfort, a substantial number of parents acknowledged the importance of equipping youth with accurate sexual health information, though they differed on who should deliver it and how.

Therefore, a study on the role of trusted adults in seeking healthcare and access to information related to health among adolescents is important, given that the said population has reportedly less access to such information, especially sensitive topics such as sexual health [20] and mental health [21]. A previous study from India [22] showed that adolescents relied much on social media for such essential information, which is prone to carry misinformation [23]. In the absence of reliable adult guidance or formal information channels, adolescents are left to navigate complex health concerns in isolation, with potentially adverse outcomes.

Against this backdrop, our study aims to empirically examine healthcare access and health information exposure among urban adolescents in India. Drawing from an online survey conducted during the COVID-19 pandemic, a period of exceptional disruption, we explored how adolescents navigated their health needs amid mobility restrictions, heightened stress, and changing support structures. The pandemic context provided us a unique and urgent lens to understand adolescent health-related information and health-seeking behavior. It was a time when schools were closed, health systems were strained, and peer and teacher networks were physically inaccessible, potentially worsening feelings of isolation and emotional distress. Globally, studies have documented that the COVID-19 pandemic significantly worsened adolescent mental health [24]. In India, emerging evidence suggests that adolescents bore the brunt of these disruptions, often without adequate psychosocial support [25]. In such a context, the presence, or absence, of a trusted adult who could provide guidance, reassurance, or connect youth to care, becomes a significant determinant of both health access and wellbeing.

In this pretext, our study investigated the extent to which adolescents in urban India had access to healthcare and health-related information during the COVID-19 lockdown, and critically, the role played by trusted adults in shaping this access. Specifically, we focus on three dimensions of adolescent health engagement.

First, we examined whether adolescents reporting mental health symptoms had access to a healthcare provider or a trusted adult during the lockdown. Given the restrictions on physical mobility, we also explored whether they utilized telehealth services as an alternative means of seeking support.

Second, we assessed what health-related information adolescents reported receiving during the pandemic. We explored five key domains: mental health, sexual and repro- ductive health, substance use, general ailments, and COVID-19 itself. These categories represent important areas of adolescent vulnerability, where misinformation or lack of information can lead to delayed care, health risks, or stigma.

Finally, we investigated whether the presence of a trusted adult was associated with improved outcomes across the above dimensions. We asked: Did adolescents who identified having a trusted adult in their lives report greater access to a healthcare provider or utilizing telehealth services during the lockdown? Were they more likely to have access to health-related information? And did they show better mental wellbeing compared to peers who reportedly lacked such support? Grounded in existing literature that highlights the protective effects of adult support on adolescent health, we hypothesized that adolescents with access to a trusted adult would fare better across all indicators. Specifically, we expected this group to report a greater access to healthcare providers, greater access to all relevant health information and topics, and lower levels of psychological distress.

With the aim to address the above questions, our study builds on a growing body of work that situates adolescent health outcomes not merely within the infrastructure of care delivery, but within relational ecosystems of trust and support. It also contributes a novel, pandemic-era perspective from urban India. By centering the role of trusted adults, this research reimagines the importance of relational capital in public health strategies targeting adolescents and offers policy-relevant insights for designing more responsive, youth-centered healthcare systems.

## 2 Methods

### 2.1 Study Design

We employed a cross-sectional design using an online survey administered to adolescents in India during the second wave of the COVID-19 pandemic, between May and October 2021. The survey aimed to assess mental health, access to healthcare and health-related information, and the presence of trusted adults among adolescents during this period of heightened vulnerability. Given the constraints of the pandemic and the lockdown, data collection was conducted entirely online. Online surveys have been widely utilized given the challenges in data collection during the COVID-19 pandemic, globally [26]. The target population comprised adolescents aged 10 to 19 years who were enrolled in grades 5 to 12 across government, government-aided, and private schools in urban areas of India. A convenience sampling approach was adopted. Participants were recruited through networks of participating research institutions, Lady Hardinge Medical College in New Delhi and Sri Ramachandra Institute of Higher Education and Research in Chennai, as well as through community-based organizations and affiliated school networks. School authorities provided approval for survey dissemination, and survey links were shared with students by either school staff or research team members, depending on institutional preference. Due to the decentralized and network-based dissemination strategy, a formal response rate could not be calculated. Nevertheless, dissemination across ten schools and multiple community platforms brought some geographic and demographic diversity in the sample.

### 2.2 Survey Instruments and Measures

The online survey was administered in English, Hindi, and Tamil. It included validated tools and structured questions on key domains relevant to adolescent health and well- being. Mental health was assessed using the validated Patient Health Questionnaire-4 (PHQ-4) [27], a brief screening tool for anxiety and depression. Participants were asked to complete the PHQ-4 twice: once reflecting their mental health status during the second wave of COVID-19 (Cronbach’s alpha was 0.78), and once retrospectively for the period preceding the second wave (Cronbach’s alpha was 0.70). Additional items captured information on access to healthcare providers and telehealth services, frequency of healthcare-seeking behavior, and whether the adolescent had a trusted adult to approach for any health-related concern. As part of the survey, we also asked adolescents whether, in the past year, any medical provider, teacher, school counselor, or other professional had provided them with information or personally discussed a range of health-related topics. These included mental health, reproductive and menstrual health, nutrition-related issues, gender and sexuality, substance use, COVID-19, and other general medical concerns. The survey was reviewed and pilot-tested with a small group of adolescents to assess comprehension, feasibility, and overall functionality. Feedback from this group was used to revise and finalize the instrument.

### 2.3 Statistical Analyses

All statistical analyses were conducted using Stata version 18. We first generated de- scriptive statistics to summarize participant characteristics and key variables of interest, including age, gender, mental health status, healthcare and healthcare information access, and presence of a trusted adult in the adolescents’ lives. Categorical variables were reported as frequencies and percentages. To examine the association between the presence of a trusted adult and adolescent mental health outcomes, we conducted multivariable logistic regression analyses. The primary outcome was high psychological distress, defined as a score of 6 or greater on the PHQ-4 scale [28]. The key independent variable was the presence of a trusted adult (yes/no). All models were adjusted for age (continuous), gender (male/female), and type of school (government/government-aided/private/not attending) to account for potential confounding. We estimated additional logistic regression models to explore associations of presence of trusted adults with access to professional healthcare providers, access to health-related information, and the use of telehealth services. These models were similarly adjusted for age and gender. Statistical significance for all models was assessed at an alpha level of 0.05. Cases with missing values on any of the variables included in the regression models were excluded using listwise deletion. No imputation was performed.

### 2.4 Ethical Considerations

Participation in the study was entirely voluntary, and no monetary compensation was offered. For adolescents under 18 years of age, informed consent was obtained from their parents or legal guardians, followed by youth assent electronically through the online form which provided a written script of the consent form. Participants aged 18 and above provided direct informed consent electronically through the same online form. Prior to participation, all potential respondents were provided with a detailed study information written script embedded within the online survey platform. This script explained the study’s purpose, procedures, potential benefits, and risks, and clearly stated that participation was voluntary and could be withdrawn at any time without penalty. The script also emphasized that responses would remain anonymous and de-identified, and that no personally identifying information would be shared publicly or outside the research team. Physical or paper-based written consent was not feasible due to the online nature of the survey and the ongoing COVID-19 pandemic lockdowns. However, both participants and parents (in the case of minors) were provided access to this consent form and were required to electronically sign and provide consent and/or assent prior to participation.

All responses were stored securely, and strict confidentiality measures were followed. Access to the de-identified data was limited to authorized members of the research team. Privacy was safeguarded by ensuring that no names, contact information, or personally identifiable data were linked to survey responses. The study adhered to all relevant ethical guidelines and received approval from the ethics committees of University of Washington, Seattle, USA (00011878 and 00012011), Lady Hardinge Medical College, New Delhi, India (LHMC IEC 2020/24 and LHMC IEC 2021/24) and Sri Ramachandra Medical College Chennai, Tamil Nadu, India (IEC-NI/20/July/75/48)(ICMR approval numbers 2020–9678 and 2020–9679)..

## 3 Results

### 3.1 Sample Characteristics

Our analytical sample comprised a total of 790 adolescents aged between 10 and 19 years. A higher proportion of participants was in early to mid-adolescence: 53.67% were aged 13–15 years, followed by 21.39% aged 10–12 years, 18.73% aged 16–17 years, and 6.20% aged 18–19 years. In terms of gender, 62.91% of the respondents were female. We also found that 30% of the adolescents in our sample reported high levels of psychological distress. In terms of schooling, the vast majority (92.41%) of adolescents were attending private schools, while smaller proportions were enrolled in government (4.05%) and government-aided schools (2.78%), and a very small share (0.76%) reported not attending school at the time of the survey.

### 3.2 Access to Healthcare

In exploring the adolescents’ access to healthcare, we first assessed how frequently they reported visiting a healthcare provider such as a doctor or nurse. Among the full sample, 44.81% reported visiting a provider only once a year, 35.70% reported multiple visits in a year, and 19.49% said they never visited a provider (Table 1).

Owing to the fact that adolescents in the full sample may not always require to visit any healthcare provider, we also assessed this among the subset of adolescents who reported having mental health distress as measured by the PHQ-4. We found that the distribution was largely similar: 43.46% reported one visit per year, 38.82% reported multiple visits, and 17.72% had no visits.

Access to a professional healthcare provider (such as a doctor, nurse, or counselor) was reported by a substantial 63.04% of adolescents, overall. Even among those who reported high psychological distress, this proportion was similar (62.03%). Telehealth use also remained limited. Only 32.41% of adolescents overall had accessed services via phone or video. Despite increased digital engagement during the pandemic, two-thirds of adolescents, including those with distress, had not used telehealth as a mode of care.

However, one of the most encouraging findings was that support from trusted adults appeared to be relatively strong across the sample. A large majority (85.32%) reported having a trusted adult in their life, such as a parent, guardian, or other figure. Still, among those with mental health symptoms, this number was lower at 76.37%, highlighting that nearly one in four adolescents experiencing distress lacked a dependable adult relationship.

### 3.3 Access to Health-Related Information

Our findings also highlighted the types of health information adolescents received during the pandemic. Information about COVID-19 was the most available, with 71.9% of adolescents reporting that a professional, such as a teacher, counselor, or healthcare provider, discussed the topic with them. Mental health followed, with 58.6% receiving such information (Table 2). Access to nutrition-related information, such as anemia, was reported by 52.5% of respondents, while only 47.2% received information on menstrual health. In contrast, more stigmatized or sensitive topics saw much lower engagement: only 29.5% received information on reproductive health, and even fewer had access to discussions on gender and sexuality (32.1%) or substance use (32.9%). These figures highlight a missed opportunity to equip adolescents with comprehensive knowledge around health and healthcare during a time of heightened vulnerability.

While our study did not ask adolescents to identify the specific individuals they personally turn to for health information, we did explore the broader landscape of where they, and peers their age, typically receive information related to topics such as mental health, menstrual and reproductive health, substance use, and gender identity. Digital platforms were the most frequently cited source, with nearly 59% of adolescents saying they accessed health-related information through the internet or social media (Table 3). Family members also remained an important source of information, with about 57% of adolescents indicating that they received guidance from parents or other relatives. Peer networks were also prominent, with 47.5% of respondents citing friends as key sources. Schools also served as a medium of health information, as reported by about 50% of adolescents. However, more traditional forms of media like television (36.9%) and newspapers (26.6%) still played a role, though to a lesser extent, likely reflecting generational shifts in media consumption preferences.

Notably, health professionals such as doctors and nurses were mentioned by only 24.1%, and very few adolescents reported receiving information from counselors (0.5%). These figures indeed highlight a significant gap between adolescents and formal support systems, especially concerning given the reliability and quality such sources can offer.

### 3.4 The Role of Trusted Adults in Adolescent Health and Health- care

We finally ran a series of multiple logistic regressions to assess the influence of having a trusted adult on several healthcare and health-related outcomes among adolescents in our study. We found that adolescents who reported having a trusted adult had significantly higher odds of reporting access to a professional healthcare provider (AOR = 1.98, *p* = 0.001), controlling for age, gender, and type of school attended (Table 4). However, there was no significant association between having a trusted adult and use of telehealth services such as phone or video consultations (AOR = 1.07, *p* = 0.753).

In terms of access to health-related information, adolescents who had a trusted adult in their lives had higher odds of reporting that they received information on mental health (AOR = 2.38, *p* < 0.001), nutrition (AOR = 1.79, *p* = 0.005), accidents and injuries (AOR= 1.89, *p* = 0.002), and COVID-19 (AOR = 1.91, *p* = 0.003). These associations were statistically significant after adjusting for age, gender, and school type. By contrast, the presence of a trusted adult was not significantly associated with receipt of information on more sensitive health topics such as reproductive health (AOR = 1.43, *p* = 0.129), gender and sexuality (AOR = 1.23, *p* = 0.359), menstrual health (AOR = 1.48, *p* = 0.061), or substance use (AOR = 1.25, *p* = 0.322). While the odds ratios in each case indicated a positive direction of association, the results were not robust to statistical significance.

We also assessed if having a trusted adult resulted in the likelihood of better mental health outcomes among the adolescents. As hypothesized, we found that adolescents who had a trusted adult had significantly lower odds of experiencing high levels of psychological distress. During the COVID-19 period, the odds of reporting high mental health symptoms were 60% lower among those with a trusted adult compared to those without (AOR = 0.40, *p* < 0.001) (Table 5). Similarly, prior to the pandemic, adolescents with a trusted adult had 57% lower odds of high distress (AOR = 0.43, *p* < 0.001), as they recalled. These associations highlight the protective influence of trusted relationships during both crisis and non-crisis contexts.

## 4 Discussion

### 4.1 Findings

The findings from our study, involving 790 adolescents, shed light on how adolescents in urban India navigated healthcare access and information during the COVID-19 pandemic, and how the presence of a trusted adult helped them access healthcare and health-related information. Most importantly, our study found that adolescents who had a trusted adult in their lives were more likely to report having access to a healthcare provider, receiving important health information, particularly about mental health, nutrition, and COVID-19, and had significantly lower chances of experiencing high psychological distress. However, the study also revealed important gaps. While adolescents did receive information on health, much of it came from informal sources like family, friends, and digital media. Only a very few adolescents reported receiving information from professionals such as doctors, nurses, or counselors.

The first objective of our study was to assess access to healthcare providers and services among adolescents. About 40% of our sample reported not having access to a healthcare provider whom they could reach out to in case of issues related to their physical or mental health. What is concerning is that about 17% of adolescents in the sample who reported psychological distress during the second wave of the COVID-19 pandemic did not access any formal healthcare services. Global literature discusses the multitude of challenges among adolescents in accessing healthcare services [29]. For instance, legally, they often require parental or marital consent, and provider biases can further restrict access to essential services, especially for sexual and reproductive health [30]. In fact, younger adults also experience greater financial burden in accessing healthcare compared to older adults. Further, adolescents also seek privacy and confidentiality in accessing healthcare, especially for sensitive issues such as sexual and reproductive health or mental health. They fear legal procedures that may require informing their parents when the need arises [31]. This demands greater equity in healthcare services provided for adolescents, as emphasized by the WHO framework on principles of adolescent-friendly health clinics [32].

In India, the quality of such clinics, however, remains low. An assessment carried out by Santhya et al. [33] showed surprisingly low awareness of Adolescent-Friendly Health Clinics (AFHCs) among adolescents and youth, with about 5% of young men and 8% of young women reportedly being aware of AFHCs that were, in fact, located only within a radius of 5–10 kilometers. More concerning was that the study found that less than 1% of such young men and women ever accessed the AFHCs [33]. The same study also discussed low utilization of formal healthcare services in case of mental health challenges. Their data showed that only 0.8% of males and none of the females approached community health workers, and minimal engagement was seen with government medical officers. Instead, a significant percentage, 39.1% of males and 14.4% of females, visited private providers, while informal providers were consulted by 41.1% of males and 43.3% of females. Friends, family, and peers were also notable sources of support, particularly for young women, highlighting a preference for more confidential and less stigmatizing avenues of care. These statistics, which echo in our findings to an extent, highlight the persistent social and infrastructural challenges in adolescent healthcare in India.

We then explored the extent to which adolescents in our sample had access to crucial health-related information. Despite the study being conducted during the second wave of COVID-19, about 30% reportedly did not receive any information from a professional on COVID-19 and about 40% reported not receiving information on mental health. Previous research has argued that mental health is underexplored in India, compounded by the socioeconomic landscape that hinders discussions around it [34]. We also found that information on stigmatized topics such as sexual and reproductive health, gender and sexuality, and substance use was much lower, highlighting disparities in health-related information likely due to prevailing stigma around such topics. This is concerning since previous large-scale studies from selected sites in India have highlighted low levels of awareness around these topics among adolescents and young adults, with only 8.7%, 11.4%, and 6.6% having adequate knowledge of sexual intercourse and pregnancy, contra- ceptive methods, and HIV/AIDS, respectively [35]. Mental health literacy among Indian adolescents is also reportedly low [21]. Due to cultural norms, stigma around discussing such topics, and judgmental attitudes of healthcare providers (the reliance on professional healthcare providers in our study was extremely low), adolescents and younger adults often relied on informal sources for such information. In our study, we found substantial reliance on digital media for information on physical and mental health, likely because these avenues were more private for the adolescents. A previous study from India has shown that exposure to social media was associated with greater access to such sensitive information around sexual and reproductive health [35]. However, information from such informal sources could carry unreliable data [23]. What was encouraging in our findings is that more than 50% of the sample reported accessing information on health from family members and schools. Adult figures from family and school are often the first point of contact for adolescents and can become a source of trust while accessing health-related information and guidance.

Therefore, we finally assessed if having a trusted adult in the adolescents’ lives had any influence on their healthcare access and access to health-related information. Trusted adults play an important role in adolescent health, particularly in promoting access to care and fostering mental well-being [36]. Findings from our study support this, highlighting that adolescents with a trusted adult in their lives are more likely to report having access to a healthcare provider.

However, a limitation observed in our study is the absence of a significant association between trusted adult presence and telehealth usage. This, in fact, may be reflective of structural barriers, including limited digital access, low e-health literacy, and inadequate infrastructure, especially in rural or low-income settings. Studies by Nanda et al.[37] and Rea et al. [38] argue that telemedicine services remain underutilized among Indian adolescents due to a lack of familiarity and trust in virtual care platforms.

We also found that adolescents who reported having a trusted adult were more likely to be better informed about general health issues such as mental health, nutrition, and accident prevention. Parents and trusted adults have been found to be sources of health information among youngsters in many studies [39, 40]. When adolescents place trust on adults, their proximity to them likely increases. In healthcare resource-poor settings, and when awareness about health is low, adolescents could reach out to such trusted adult figures in their lives for health-related information. Given that mental health literacy and awareness in India is poor, the protective role of having a trusted adult on access to information on mental health is an important finding, even from a policy perspective.

While we did not assess who these trusted adults were, we found that more than 40% of adolescents reportedly relied on family members for health-related guidance. There is a need to understand further what communication patterns exist between these trusted adults and the adolescents and if there are any barriers in such communication. This is important because we could not find any statistically robust evidence (although positive) on the association of having a trusted adult and access to sensitive health-related information such as sexual and reproductive health, menstruation, and substance use. Despite the trust adolescents placed in some adults, our findings showed no significant improvement in their access to such information. This is consistent with findings from Likith [41], who reported that cultural stigma and discomfort often prevent open dialogue on sexual and reproductive health between adolescents and even their most trusted adults in Indian contexts. Norms around modesty, fear of encouraging perceived deviance, and lack of parental knowledge could all contribute to this communication gap. Studies have also discussed how parents felt it was challenging for them to even discuss weight-related issues with their children [42]. Since the role of having a trusted adult in access to health information on non-sensitive topics as well as mental health is evident, programmatic and social efforts in reducing the gap in communication about sensitive topics could make the protective factor of having a trusted adult more robust in other dimensions. In fact, previous research, including a meta-analysis across three decades, suggests that parent and adolescent SRH communication was associated with safer sexual behavior and choices among adolescents [43]. Furthermore, structured institutions such as schools can offer avenues for adolescents to establish and interact with “trusted adults” who are non-familial. In fact, in a study from India, adolescents have felt the need to have more discussions around SRH in school settings [44]. Having empathetic teachers and counsellors in schools, who could potentially become trusted adults for adolescents, can mitigate a significant gap in accessing SRH-related information.

One of the most important findings of our study is the contribution of trusted adults in reducing psychological distress among adolescents. In times of crisis, such as the COVID-19 pandemic and beyond, these relationships appeared particularly protective, echoing findings from global studies that show supportive adult relationships can buffer the mental health and emotional effects [45, 16]. Trusted adults offer emotional support, guidance, and stability, resources adolescents may otherwise lack, especially in low-resource or disrupted environments.

In fact, previous research with eleventh-grade students has shown that such relationships with trusted or significant adults were of extremely high quality [46]. These relationships not only play a supportive role in overall mental well-being but also help adolescents in their academics [45, 14]. In this context, the study by Meltzer et al.[14] discussed the perspectives of trusted adults of adolescents, who confirmed that their presence offers “support, encouragement, role modelling and practical assistance in a low-key, direct and non-hierarchical way.”

### 4.2 Implications

The findings of our study offer several important implications for policy and practice aimed at improving adolescent health in India. One of the most urgent challenges is ensuring that adolescents have access to a healthcare provider. This is consistent with broader research showing that adolescents globally face legal, structural, and social barriers to accessing care. Parental consent requirements, judgmental attitudes of providers, and confidentiality concerns disproportionately affect adolescents seeking support for sensitive issues. Addressing these barriers requires training healthcare providers in empathetic, non-judgmental care practices. Several previous studies discussed interventions on making healthcare providers ready for adolescent-friendly health services positively [47], which in the Indian context too holds much promise. Adolescents’ lack of access to healthcare professional, despite their reporting of psychological distress could also be due to the lack of mental health awareness among such adolescents. Previous studies have shown that a greater mental health literacy was associated with greater mental health attitudes and health-seeking intentions [48]. However, there is a lack of systemic, inclusive, and accurate mental-health education in schools. Therefore, interventions [49, 50], which showed promise of enhancing mental health literacy should be scaled up and institutionalized. This must be co-designed with youth input and implemented alongside confidential support systems, such as trained school counselors. There should also be a stronger research focus on understanding the current state of mental health literacy among adolescents in India. At present, large-scale surveys such as the National Mental Health Survey, mostly focuses on estimating mental health prevalences, however, equally important is the understanding of how adolescents’ (as well as adults’) awareness about mental health related symptoms.

Most notably, we found that the presence of a trusted adult significantly enhanced adolescents’ access to healthcare providers and information on general health topics as well as mental health and nutrition. Therefore, a critical area of intervention could be the formalization and support of trusted adults in adolescent lives. First, it is important to understand who adolescents perceive as a trusted adult, what communication dynamics do exist in such dynamics. We could not locate much data related to this, apart from a few studies which focused on parent-adolescent communication. Our finding, which highlight that adolescents with a trusted adult are more likely to have access to a healthcare provider and access to crucial health-information, demands a more rigorous inquiry in understanding the concept of trusted adult. This could also help us better understand if there are any barriers to such communication, especially around sensitive topics such as sexual and reproductive health. Second, potential of exploiting the positive influence of trusted adults could also remain underleveraged due to the lack of clear guidelines or training frameworks. Meltzer et al. [14] emphasize that trusted adults should offer support in a genuine, empathetic, and non-hierarchical way. Programs could benefit from developing structured training modules for community workers, teachers, and parents to become effective trusted adults, especially in resource-poor or at-risk settings. NGOs can play a central role in both capacity-building and direct service delivery. Thara and Patel [51] note that NGOs are often better positioned than government actors to build trust, particularly in mental health and SRH domains. Their community presence allows them to bridge institutional gaps and create adolescent-responsive services. Interventions such as the SAMA project in India [52] have already started testing how partnerships between schools and NGOs can enhance adolescent anxiety management and health literacy through school systems.

To ensure adolescents have access to healthcare and credible information, it will require a multi-pronged approach such as training empathetic providers, integrating health education into school curricula, supporting trusted adults through structured frameworks, and leveraging NGO expertise. These steps, when taken together, has the potential to reduce the reliance on informal or potentially unreliable sources, enhance adolescent well-being, and build a more resilient healthcare ecosystem for India’s youth.

### 4.3 Limitations and Strengths

This study has several limitations that should be considered cautiously when interpreting the findings. First, the online, cross-sectional design and use of a convenience sample limit both the generalizability of results and the ability to draw causal inferences. Online surveys, particularly during the COVID-19 pandemic, have been criticized for issues such as selection and response bias, participant fatigue or disinterest, and concerns about data validity due to possible fraudulent responses. Earlier studies have noted these challenges, emphasizing the need to carefully document the methodology and ethical precautions specific to online data collection [53]. To mitigate some of these concerns, we distributed the survey through school authorities, individuals whom adolescents were likely to trust. We also provided clear instructions about the study’s purpose and emphasized confidentiality and anonymity to encourage honest responses. Nonetheless, the lack of a probabilistic sampling frame restricts the study’s representativeness.

Second, our sample largely comprised adolescents from urban neighborhoods and private schools, groups that typically enjoy better access to digital infrastructure, educational resources, and healthcare compared to their rural counterparts. Despite these relative advantages, many still reported lacking access to a healthcare provider, which is concerning. Given the documented disparities in healthcare access and quality in rural India, we hypothesize that the situation could be worse among rural adolescents; however, our study design does not allow us to make this claim empirically. Finally, our operationalization of “trusted adult” was conceptually broad. Due to limitations in survey length and respondent burden, we did not probe who these trusted adults were, whether parents, teachers, extended family, or others. A more detailed understanding of these relationships could have yielded deeper insights into the role and influence of different adult figures in adolescents’ lives. Nevertheless, our study offers a strong foundation for future research on this topic, and the associations found are both promising and policy relevant.

A key strength of this study is its novel focus on the role of trusted adults in adolescent healthcare and health-information access and mental well-being in the Indian context. Despite its limitations, it is among the first empirical efforts to explore how the presence of a trusted adult influences health-seeking behavior and access to health information among adolescents in urban India. This contribution is significant, as it opens new avenues for designing interventions that leverage existing social support systems, particularly family and school environments, to improve adolescent health outcomes. The findings, though preliminary, highlight the promise of incorporating trusted adult frameworks into adolescent health programming and policy.

## 5 Concluding Remarks

This study highlights the critical, yet often overlooked, role of trusted adults in shap- ing adolescent health outcomes in urban India. While much attention has been paid to infrastructure and service delivery, our findings suggest that relationships of trust and emotional safety are equally important in determining whether adolescents access healthcare and reliable information, especially during times of crisis like the COVID-19 pandemic. Adolescents with a trusted adult were more likely to seek professional care, receive health-related information, and report lower psychological distress. However, gaps remain, particularly in communication around sensitive topics such as sexual and reproductive health. These findings point to the need for adolescent health policies to move beyond service provision and actively foster supportive environments, at home, in schools, and in communities, where adolescents can engage with trusted, non-judgmental adults. Strengthening these relational ecosystems could be a key step toward building a more inclusive, responsive, and youth-centered public health system.

## Figures and Tables

**Table 1:** Access to Healthcare Resources and Trusted Adults Among Adolescents

Measure	Category	Freq.	Percent
*Frequency of Healthcare Visits (All Adolescents)*			
	Multiple times a year	282	35.70
	Never	154	19.49
	Only once a year	354	44.81
*Frequency of Healthcare Visits (Mental Health Distress Subgroup)*			
	Multiple times a year	92	38.82
	Never	42	17.72
	Only once a year	103	43.46
*Access to Professional Healthcare Provider*			
	No	292	36.96
	Yes	498	63.04
*Access to Professional Healthcare Provider (Mental Health Distress Subgroup)*			
	No	90	37.97
	Yes	147	62.03
*Use of Telehealth Services*			
	No	534	67.59
	Yes	256	32.41
*Use of Telehealth Services (Mental Health Distress Subgroup)*			
	No	157	66.24
	Yes	80	33.76
*Access to Trusted Adult*			
	No	116	14.68
	Yes	674	85.32
*Access to Trusted Adult (Mental Health Distress Subgroup)*			
	No	56	23.63
	Yes	181	76.37

*Note*: Mental health distress subgroup defined by PHQ-4 scores indicating elevated distress.

**Table 2:** Access to Health Information Among Adolescents by Topic

Health Information Topic	Response	Freq.	Percent
Mental Health	No	327	41.39
	Yes	463	58.61
Reproductive Health (STIs, early pregnancy)	No	557	70.51
	Yes	233	29.49
Menstrual Health	No	417	52.78
	Yes	373	47.22
Nutrition-Related Medical Issues (e.g., anemia)	No	375	47.47
	Yes	415	52.53
Gender and Sexuality (incl. LGBTQ+)	No	536	67.85
	Yes	254	32.15
Accidents and Injuries	No	401	50.76
	Yes	389	49.24
Substance Use	No	530	67.09
	Yes	260	32.91
Other Medical Ailments or Illnesses	No	430	54.43
	Yes	360	45.57
COVID-19	No	222	28.10
	Yes	568	71.90

*Note*: Figures are based on adolescents self-reporting in a multiple checkbox type question.

**Table 3:** Sources of Health Information Among Adolescents

Source of Information	Response	Freq.	Percent
Doctor/Nurse	No	600	75.95
	Yes	190	24.05
Family	No	339	42.91
	Yes	451	57.09
Friends	No	415	52.53
	Yes	375	47.47
Television	No	498	63.04
	Yes	292	36.96
Newspapers	No	580	73.42
	Yes	210	26.58
Counselor	No	786	99.49
	Yes	4	0.51
Internet Sources	No	324	41.01
	Yes	466	58.99
Schools	No	396	50.13
	Yes	394	49.87

**Table 4:** Association of Having a Trusted Adult with Health (and related) Outcomes (Adjusted Odds Ratios)

Health Outcome	AOR	95% CI	p-value
*Predictor: Having a Trusted Adult (Yes vs No)*			
Access to Healthcare Provider	1.98	1.33–2.96	0.001
Telehealth Use	1.07	0.70–1.64	0.753
Received Mental Health Info	2.38	1.58–3.57	<0.001
Received Reproductive Health	1.43	0.90–2.26	0.129
Info Received Gender/Sexuality	1.23	0.79–1.90	0.359
Info Received Nutrition Info	1.79	1.19–2.68	0.005
Received Menstrual Health	1.48	0.98–2.23	0.061
Info Received Substance Use Info	1.25	0.81–1.93	0.322
Received Accident/Injury Info	1.89	1.25–2.85	0.002
Received Other Ailments Info	1.41	0.94–2.12	0.095
Received COVID-19 Info	1.91	1.25–2.92	0.003

*Note*: Models are adjusted for gender, age, and type of school attended. Lower odds ratios indicate lower likelihood of high psychological distress among adolescents with a trusted adult. AOR = Adjusted Odds Ratio; CI = Confidence Interval.

**Table 5:** Association of Having a Trusted Adult with Mental Health Outcomes (Adjusted Odds Ratios)

Mental Health Outcome	AOR	95% CI	p-value
*Predictor: Having a Trusted Adult (Yes vs No)*			
Psychological Distress (During COVID-19)	0.40	0.26–0.60	<0.001
Psychological Distress (Before COVID-19)	0.43	0.27–0.67	<0.001

*Note*: Models adjusted for gender, age, and type of school at- tended. Lower odds ratios indicate lower likelihood of high psychological distress among adolescents with a trusted adult. AOR = Adjusted Odds Ratio; CI = Confidence Interval.

## Data Availability

Due to the sensitive nature of the data collected from adolescent participants, the data cannot be shared publicly to ensure the privacy and confidentiality of respondents. However, aggregated or anonymized datasets may be made available upon reasonable request and with appropriate justification by contacting the corresponding author at [anupam.sharma@iitgn.ac.in].
